# Mechanistic insights into chemical and photochemical transformations of bismuth vanadate photoanodes

**DOI:** 10.1038/ncomms12012

**Published:** 2016-07-05

**Authors:** Francesca M. Toma, Jason K. Cooper, Viktoria Kunzelmann, Matthew T. McDowell, Jie Yu, David M. Larson, Nicholas J. Borys, Christine Abelyan, Jeffrey W. Beeman, Kin Man Yu, Jinhui Yang, Le Chen, Matthew R. Shaner, Joshua Spurgeon, Frances A. Houle, Kristin A. Persson, Ian D. Sharp

**Affiliations:** 1 Joint Center for Artificial Photosynthesis, Lawrence Berkeley National Laboratory, 1 Cyclotron Road, Berkeley, California 94720, USA; 2 Chemical Sciences Division, Lawrence Berkeley National Laboratory, 1 Cyclotron Road, Berkeley, California 94720, USA; 3 Walter Schottky Institut and Physik Department, Technische Universität München, Am Coulombwall 4, 85748 Garching, Germany; 4 Joint Center for Artificial Photosynthesis, California Institute of Technology, 1200 East California Boulevard, Pasadena, California 91125, USA; 5 Division of Chemistry and Chemical Engineering, California Institute of Technology, 1200 East California Boulevard, Pasadena, California 91125, USA; 6 Molecular Foundry, Lawrence Berkeley National Laboratory, 1 Cyclotron Road, Berkeley, California 94720, USA; 7 Materials Sciences Division, Lawrence Berkeley National Laboratory, 1 Cyclotron Road, Berkeley, California 94720, USA; 8 Energy Technologies Area, Lawrence Berkeley National Laboratory, 1 Cyclotron Road, Berkeley, California 94720, USA; 9 Materials Science and Engineering, University of California, Berkeley, 210 Hearst Memorial Mining Building, Berkeley, California 94720, USA

## Abstract

Artificial photosynthesis relies on the availability of semiconductors that are chemically stable and can efficiently capture solar energy. Although metal oxide semiconductors have been investigated for their promise to resist oxidative attack, materials in this class can suffer from chemical and photochemical instability. Here we present a methodology for evaluating corrosion mechanisms and apply it to bismuth vanadate, a state-of-the-art photoanode. Analysis of changing morphology and composition under solar water splitting conditions reveals chemical instabilities that are not predicted from thermodynamic considerations of stable solid oxide phases, as represented by the Pourbaix diagram for the system. Computational modelling indicates that photoexcited charge carriers accumulated at the surface destabilize the lattice, and that self-passivation by formation of a chemically stable surface phase is kinetically hindered. Although chemical stability of metal oxides cannot be assumed, insight into corrosion mechanisms aids development of protection strategies and discovery of semiconductors with improved stability.

## Abstract (web summary)

Metal oxide semiconductors are promising materials for solar energy capture but can suffer from stability problems. Here, the authors present a methodology for evaluating corrosion mechanisms and apply it to BiVO_4_, revealing chemical instabilities that are not predicted from thermodynamic considerations alone.

## Graphical abstract

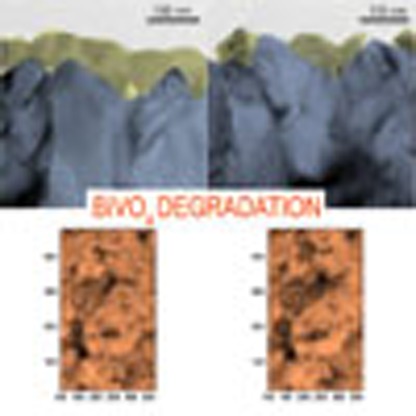

Photoelectrochemical conversion of solar to chemical energy is an attractive approach for sustainable production of hydrogen and hydrocarbon fuels^1^,^2^. However, harnessing light to split water involves thermodynamic and kinetic challenges, with major efficiency losses associated with oxygen production^1^,^2^,^3^. Furthermore, material stability is often poor under the harsh conditions in which this reaction is performed. Therefore, much attention has been devoted to the development of *n*-type thin film semiconductors that are chemically stable, can efficiently harvest sunlight and can oxidize water when coupled to appropriate catalysts^3^. Among available candidates for photoanodes, metal oxides are a promising class of potentially durable materials, as they are usually less readily oxidized than water^3^,^4^. However, many of the most stable metal oxide semiconductors possess wide bandgaps that render them impractical for solar energy conversion. Thus, a central challenge is the discovery and development of new semiconductors that are stable and have bandgaps suitable for efficiently capturing solar energy. In recent times, the quest for such materials has been accelerated by advances in high-throughput computation for prediction of electronic structure and thermodynamic stability of semiconductors^4^. Understanding the present limits of such computational approaches and improving their ability to predict the complex behaviours of real materials requires direct comparison with experiments using known semiconductors.

Although thermodynamic considerations provide a starting point for evaluating (photo)electrochemical stability of materials under the conditions present in solar fuel generators, kinetic factors can also play a dominant role on achievable operational durability. Indeed, some materials exhibit chemical instabilities that cannot be explained by thermodynamic considerations alone. One such material is BiVO_4_, which has been actively investigated as one of the highest performance oxide photoanodes for water splitting^5^. This material has garnered significant interest, because it absorbs in the visible range, with a bandgap of 2.5 eV (ref. 6), and has conduction and valence band positions—∼4.77 and 7.27 eV below the vacuum level, respectively—that are well-suited for generating large photovoltages and driving the oxygen-evolution reaction (OER)^7^. The calculated oxidation potential of BiVO_4_ has been predicted to be slightly more positive (1.24 V versus reversible hydrogen electrode, RHE) than the water oxidation potential (O_2_/H_2_O, 1.23 V versus RHE), suggesting that this material could be resistant to photoinduced corrosion under water splitting conditions^4^. In contrast to this prediction, this material suffers from photoelectrochemical instability in both near-neutral and alkaline pH ranges^8^,^9^,^10^. Although photocorrosion of BiVO_4_ has been correlated with vanadium leaching from the surface into the electrolyte solution^9^,^11^,^12^, the mechanisms of photodegradation and the associated chemical transformations have not yet been identified. This poor stability undermines its practical use in large-scale integrated devices^8^.

In this work, we establish a methodology for systematically assessing the chemical and photochemical stabilities of semiconductors and apply it to a visible-light-absorbing semiconductor, BiVO_4_. We investigate chemical and photochemical corrosion pathways using BiVO_4_ thin films at near-neutral and at high pH, thereby simulating possible operating conditions in solar fuel devices^13^. These measurements demonstrate that BiVO_4_ is susceptible to chemical corrosion that is accelerated by illumination (photodegradation). Electron microscopy indicates that photodegradation initiates at and propagates from surfaces and grain boundaries, resulting in extensive dissolution of the films. Given the existence of thermodynamically stable bismuth oxides predicted by Pourbaix diagrams under these conditions, formation of a chemically stable surface layer was expected but not observed. Therefore, bulk dissolution of the film cannot be described by thermodynamic arguments. This finding is confirmed by *in situ* electrochemical atomic force microscopy (EC-AFM), which reveals that degradation under operating conditions occurs via dissolution of the film, starting at exposed facets of grains in polycrystalline thin films. To understand this chemical behaviour, we use computational methods to examine both the illuminated and dark stability. This analysis indicates that accumulation of photogenerated holes at the surface leads to structural destabilization and chemical attack via vanadium loss into solution. Although self-passivation of the material with bismuth oxide would be expected, thermodynamically stable phases of this oxide require significant structural transformations from the low-density V-leached state and are not accessible at room temperature. Therefore, photocorrosion results in bulk dissolution of the material. Our measurements reveal that non-equilibrium and kinetic factors dominate chemical and photochemical degradation processes for this commonly studied semiconductor material, and these factors must be considered for prediction of new materials that possess desired combinations of stability and efficiency. Furthermore, the kinetic accessibility of self-passivating surface layers, which broaden the range of available corrosion protection strategies, must be considered for engineering the interfaces of materials possessing lower intrinsic stability. These findings can be used to guide extensions of theory to consider intermediate (photo)chemical states and may provide enhanced predictive power for evaluating and interpreting the stabilities of promising new semiconductors for solar fuel applications. In addition, improved understanding of mechanisms governing photochemical stability will guide development of experimental strategies for stabilizing chemically sensitive semiconductors.

## Results

### Evaluation of photoelectrochemical stability of bismuth vanadate

Evaluating the stability of materials for photoelectrochemical water splitting requires testing under conditions that are relevant to practical devices. Although near-neutral conditions are often used for testing individual water splitting photoelectrodes and place less severe constraints on material stability relative to acidic and alkaline conditions, current integrated solar water splitting devices require either electrolyte recirculation or more extreme pH environments to eliminate severe pH gradients and ohmic losses^14^. Much work has been reported on the photoelectrochemical properties of BiVO_4_ at near neutral pH, where stabilization has been realized via integration of OER catalysts, such as cobalt phosphate (CoPi)^15^ and iron oxyhydroxide (FeO(OH))^8^,^16^,^17^. However, strongly alkaline conditions are often desirable for avoiding hydroxide ion depletion near the electrode and high overpotentials due to ohmic losses in water splitting devices that separate chemical products^14^,^18^. Fewer reports have addressed the characteristics of BiVO_4_ under alkaline conditions^9^,^10^. Notably, a dual layer TiO_2_/Ni coating was recently demonstrated to significantly impede degradation at high pH^9^. Under all testing conditions, the long-term stability of this material remains a question and understanding of the factors governing corrosion and photocorrosion may provide a means of predicting lifetime, preventing failure and improving strategies for catalyst integration. Importantly, understanding chemical and photochemical interactions of state-of-the-art materials, such as BiVO_4_, provides a basis for rational design of new materials by identifying kinetic degradation pathways that can be evaluated computationally.

Because of its different *p*Ka values, phosphate buffer allows for the study of stability at various pH conditions that are relevant for water-splitting applications, while maintaining equal ionic strength. Therefore, we chose to study the stability of BiVO_4_ using 1 M potassium phosphate (KPi) buffer at pH 6.8 and at pH 12.3 under biased and unbiased dark and illuminated (AM 1.5, 100 mW cm^−2^) conditions.

For this study, we selected undoped BiVO_4_ deposited by the spin coating method, because it allows for reproducible fabrication of large area planar thin films. As such, spin-coated BiVO_4_ is well suited for achieving reliable statistics, drawing comparisons between bulk and surface effects, and analysing the impact of degradation on surface chemistry and morphology. However, to confirm that the findings presented here are generally valid for the material synthesized via other methods, a variety of undoped and doped thin films deposited by different techniques, namely electrodeposition^16^, reactive sputtering^17^ and spray pyrolysis^19^, were also tested under similar conditions and yielded qualitatively similar stability results (Supplementary Figs 1 and 2).

Deposition by spin coating of molecular organic precursors onto fluorine-doped tin oxide (FTO) layers yields pure-phase monoclinic scheelite BiVO_4_ (Supplementary Fig. 3). Scanning electron microscopy (SEM) shows that the films are characterized by average grain sizes of 153±25 nm (Supplementary Fig. 3). Rutherford backscattering spectrometry (RBS) indicates a film thickness of ∼51.6±3.2 nm and is in excellent agreement with cross-sectional transmission electron microscopy (TEM) analysis, which reveals a thickness of ∼54 nm (Supplementary Fig. 3). In addition to thickness, RBS was used to evaluate thin film composition and revealed a stoichiometric ratio of 1:1 Bi:V. Importantly, complementary X-ray photoelectron spectroscopy (XPS) measurements indicate significant differences in surface stoichiometry compared with bulk, with a surface atomic ratio of 1.27:1 of Bi:V and an overall composition of 21.6 at.% Bi, 17.0 at.% V and 61.3 at.% O (compared with the ideal stoichiometric composition of 16.7 at.% Bi, 16.7 at.% V and 66.6 at.% O, respectively; Supplementary Table 1).

**Figure 1:**
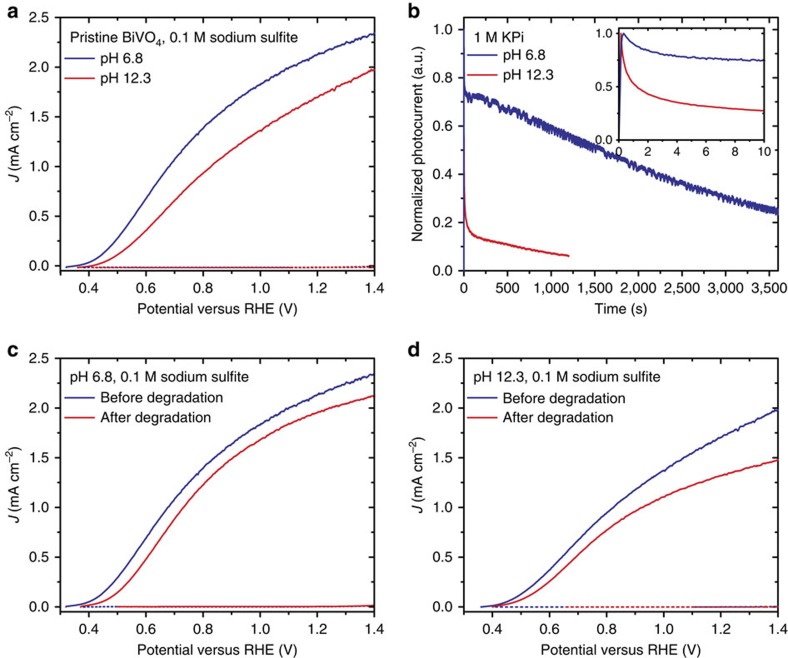
Photoelectrochemical characterization of BiVO_4_ electrodes in 1 M KPi buffer. (**a**) Photoelectrochemical *J*–*E* characterization of pristine BiVO_4_ thin films in solution containing 0.1 M sulfite at pH 6.8 (blue curve) and pH 12.3 (red curve) under light (solid curve) and dark (dotted curve). (**b**) Chronoamperometry of pristine BiVO_4_ thin films in pure KPi solution (no sulfite present) at pH 6.8 (blue curve) and pH 12.3 (red curve) at a fixed applied bias of 1.23 V versus RHE. (**c**) *J*–*E* characteristics of BiVO_4_ thin films in solution containing 0.1 M sulfite at pH 6.8 under light (solid curve) and dark (dotted curve) before (blue curve) and after (red curve) testing. (**d**) *J*–*E* characteristics of BiVO_4_ thin films in solution containing 0.1 M sulfite at pH 12.3 under light (solid curve) and dark (dotted curve) before (blue curve) and after (red curve) testing. All measurements under illumination were performed using simulated AM 1.5 light (Solar Light) adjusted to 100 mW cm^−2^ using a calibrated Si photovoltaic cell (SolarSim calibration, Newport).

As a starting point for the corrosion studies, the performance of BiVO_4_ thin films was evaluated at different pH values in the presence of 0.1 M sodium sulfite as a hole scavenger. As sodium sulfite oxidation is thermodynamically favoured and is kinetically facile with respect to water oxidation, this approach allows for determination of the charge separation and extraction efficiency in BiVO_4_, without influence by the intrinsically poor catalytic activity of its surface^8^. Figure 1a show a comparison of photocurrent density versus potential (*J–E*) anodic sweeps of pristine BiVO_4_ obtained at pH 6.8 and at pH 12.3, respectively. The sample measured at pH 6.8 before its degradation is characterized by an open circuit potential of 0.35 V versus RHE and a current density of 2.2 mA cm^−2^ at 1.23 V versus RHE. The sample measured at pH 12.3 before stability testing shows an identical open circuit potential, but exhibits a significantly reduced fill factor and a smaller photocurrent of 1.8 mA cm^−2^ at 1.23 V versus RHE.

The reduced fill factor and photocurrent density observed under alkaline testing conditions suggest that rapid chemical modification of BiVO_4_ at high pH (see below) introduces additional recombination centres at the surface and/or an insulating surface layer that increases the resistance to charge transfer across the interface^3^,^20^. Furthermore, analysis of cyclic voltammograms (CVs) reveals an increase of the *J–E* hysteresis (see Supplementary Fig. 4) at high pH. This hysteresis is consistent with surface state charging and may also be affected by modified interfacial energetics arising from changing occupancies of surface states during illuminated cycling. The pronounced hysteresis under alkaline conditions further suggests that chemical modification at high pH introduces electronically active surface states.

Chronoamperometry measurements show the change of photocurrent from BiVO_4_ thin films as a function of time at fixed applied potential of 1.23 V versus RHE at both pH 6.8 and 12.3 in the absence of hole scavenger (Fig. 1b). In both cases, these data are characterized by a rapid decrease of the photocurrent during the first ten seconds (inset). This effect is significantly accelerated under alkaline conditions and is consistent with both the reduced fill factor and increased hysteresis relative to near neutral conditions, as described above. The initial fast photocurrent decay is followed by a slower decay over the course of the 60 min (pH 6.8) and 20 min (pH 12.8) measurements.

After the 1 h chronoamperometry test in phosphate buffer at near neutral pH, cyclic voltammetry of degraded BiVO_4_ was again performed in the presence of sacrificial hole acceptor (Fig. 1c). We observe an anodic shift in the photocurrent onset potential of about 0.1 V, as well as decreases of both the current density and fill factor. Furthermore, analysis of CVs reveals that significant hysteresis is present following sustained operation (Supplementary Fig. 4). This finding suggests that consumption of the films by photocorrosion is also accompanied by the introduction of higher capacitance, probably due to charging of surface defect states, even at moderate pH.

Similarly, the 20 min chronoamperometric stability test at high pH reduces the photoelectrochemical performance of this material, with an anodic shift of the onset potential (∼0.1 V) and decrease of the current density. Hysteresis remains approximately constant, supporting the finding that chemical transformation of the surface occurs immediately on immersion in the alkaline electrolyte. Interestingly, although the photocurrents were observed to significantly decline after chronoamperometric testing at 1.23 V versus RHE in the absence of sacrificial reagent (by ∼75% at pH 6.8 and ∼95% at pH 12.3, Supplementary Fig. 5), subsequent *J–E* characterization in the presence of Na_2_SO_3_ revealed much less pronounced reductions in the photocurrent density (Fig. 1). As neither the local nor bulk pH changes can account for the observed behaviour (Supplementary Note 1), this observation suggests that, in addition to introducing electronic defect states, chemical modification of the surface also reduces the native catalytic activity of the surfaces for water oxidation.

As described below, elemental analysis suggests that photocorrosion may result in enrichment of bismuth oxide on the surface. As oxides of bismuth are known to exhibit poor catalytic activity for water oxidation^21^, chronoamperometric testing in pure phosphate buffer yields dramatic performance degradation under sustained operation. However, it is important to note that these measurements convolve catalytic activity with light capture and charge extraction efficiencies. Therefore, comparison of *J–E* curves obtained with a sacrificial hole acceptor, such as those shown in Fig. 1, more accurately represent the effects of photocorrosion and chemical modification on semiconductor performance for solar energy conversion.

### Compositional analysis of bismuth vanadate photoanodes

To obtain insights into the mechanism of photodegradation and understand the changes in photoelectrochemical characteristics under operational conditions, we performed a compositional analysis of thin films before and after the chronoamperometric testing. As described above, for the case of pristine BiVO_4_ thin films, a combination of XPS and RBS was used to analyse differences between the surface and bulk composition, which can be affected by surface chemistry and have an important impact on interfacial charge transport, as well as catalysis.

As summarized in Table 1, RBS indicates that the bulk ratio of Bi:V is stoichiometric, with a ratio of 1:1, and does not change during the degradation process. From fitting of RBS data, we calculated the thickness of the pristine BiVO_4_ layer (51.6±3.2 nm), as well as of the degraded samples (44.1±3.7 nm at pH 6.8 and 37.4±1.8 nm at pH 12.3). The progressive thinning of the layer during sustained operation under illumination indicates photocorrosion of the films over time, with nominal rates of 0.125 nm min^−1^ at pH 6.8 and 0.71 nm min^−1^ at pH 12.3 (7.5 nm h^−1^ at pH 6.8 and 42.6 nm h^−1^ at pH 12.3). Thus, photocorrosion is accelerated under alkaline conditions. However, it must be noted that the RBS model assumes a homogenous corrosion of the film, which is not supported by the SEM and TEM results (*vide infra*). In addition, quantification of bulk oxygen content by RBS in the degraded samples was complicated by the underlying FTO.

**Figure 2:**
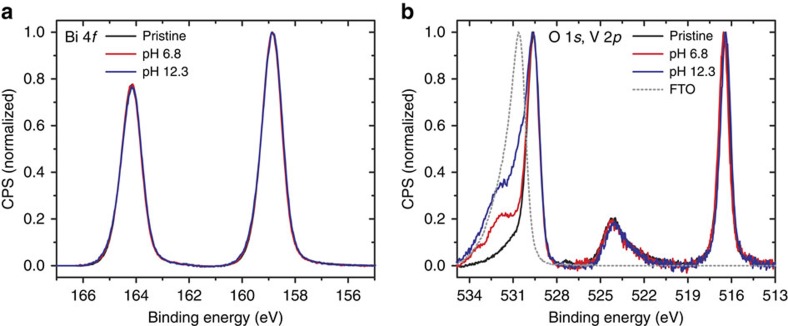
XPS spectra of pristine and degraded electrodes. (**a**) Bi 4*f*, (**b**) O 1*s* and V 2*p* core levels of pristine (black) and degraded at pH 6.8 (red) and pH 12.3 (blue) BiVO_4_ samples compared with FTO (dashed grey).

In contrast to the constant bulk stoichiometry determined by RBS, important changes of the surface composition are observed by XPS (Fig. 2). Comparison of photoemission spectra obtained before and after photoelectrochemical stability testing reveals no significant changes in the Bi 4*f* or the V 2*p* core level lineshapes. Analysis of the O 1*s* spectral region reveals a main component at 529.4±0.3 eV from oxygen in the thin film. However, a shoulder at 531.7±0.1 eV emerges following illuminated chronoamperometric testing. As discussed below, SEM and TEM analyses prove that the films become discontinuous after stability testing. Therefore, photoelectrons from regions of bare FTO contribute to the overall spectrum. Comparison with a reference FTO standard confirms this and enables assignment of the 531.7±0.1 eV component to oxygen in the FTO substrate. As summarized in Table 1, analysis of XPS core-level intensity changes shows that surfaces become enriched with Bi and slightly depleted of V following chronoamperometric testing at both pH 6.8 and 12.3. Loss of V from the surface is consistent with prior reports of BiVO_4_ composition modification under aqueous conditions^9^,^11^. However, the associated enrichment of Bi, on an atomic per cent basis, suggests the possible formation of a terminal surface of bismuth oxide, such as Bi_2_O_3_ or Bi_4_O_7_. In addition, given that the valence band of Bi_2_O_3_ is energetically deeper than that of BiVO_4_, such a layer would be expected to introduce a barrier to photogenerated hole transport across the interface and could explain the anodic shift of the photocurrent onset potential and the reduced fill factor observed from *J–E* curves after stability testing^22^. However, formation of a stable passivating film of bismuth oxide, such as Bi_2_O_3_, would be expected to impede further chemical corrosion. In contrast, we observe progressive dissolution of the films, with loss of both V and Bi, as measured by RBS. In addition, similar composition results are obtained with RBS and XPS after testing the electrodes under dark conditions at an applied bias of 1.23 V versus RHE (Supplementary Table 1).

### Determination of bismuth vanadate etching rates

Although loss of vanadium from the surface of BiVO_4_ in aqueous environments is consistent with prior reports, our results suggest more extensive (photo)chemical etching, with substantial loss of material from thin films. These findings prompted chemical analysis of the phosphate electrolytes used during stability tests, to detect thin film corrosion products.

**Figure 3:**
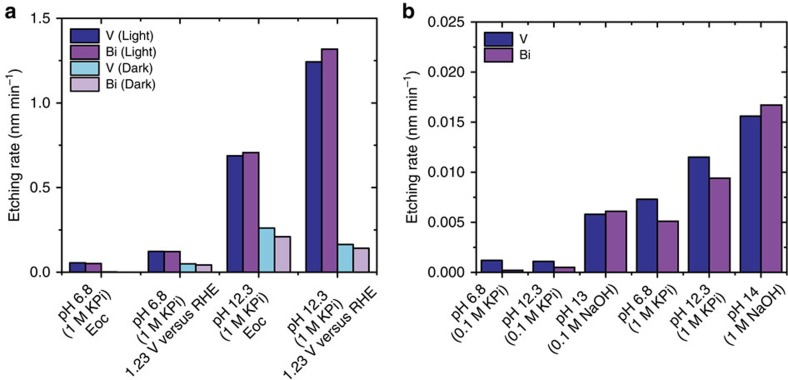
Etching rates determined by ICP-MS as a function of testing conditions. (**a**) Etching rate on the basis of V (blue) and Bi (purple) in BiVO_4_ substrates at different potentials and pH conditions under illumination and in the dark (light blue and light purple for V and Bi, respectively). (**b**) Etching rate on the basis of V (blue) and Bi (purple) in BiVO_4_ substrates soaked in the dark for 72 h in different electrolytes at different concentrations. Homogeneous thinning of films is assumed.

Using inductively coupled plasma mass spectrometry (ICP-MS), we analysed the electrolyte solutions from samples treated in different conditions. Table 2 summarizes the estimated degradation rate (nm min^−1^) on the basis of dissolved Bi and V, calculated assuming homogeneous dissolution. These results are also shown in Fig. 3 and the complete data sets and details of calculations are provided in the Supplementary Information (Supplementary Table 2 and Supplementary Fig. 6). Under all conditions, we observe loss of both Bi and V into solution. We find that BiVO_4_ degradation is accelerated, in decreasing order, by light, increasing pH and applied bias.

To better understand the role played by solution concentration, as well as by the specific use of phosphate electrolytes, different electrolytes with different concentrations were analysed and compared with the 1 M phosphate buffers (Fig. 3b and Supplementary Information). Notably, BiVO_4_ thin films soaked in different electrolytes for 72 h tend to solubilize in all of the conditions studied. However, it is clear that the phosphate buffer concentration also plays an important role in determining the degradation rate of the material. At similar pH values, we observe that higher concentration accelerates degradation (Fig. 3b). Although the origin of this concentration dependence is not currently known, trends of stability with illumination, pH and applied bias are mostly preserved (see Supplementary Table 2). Notably, for the case of samples tested in 1 M NaOH (pH 14) at 1.23 V versus RHE and under 1 sun AM1.5 illumination, the rate of vanadium loss from the films is significantly increased (Supplementary Fig. 6 and Supplementary Table 2). Indeed, under these conditions, we determine the etch rate on the basis of vanadium to be 2.7 nm min^−1^, corresponding to the entire film thickness after ∼20 min. This finding is in agreement with previous reports and is attributed to the extreme chemical instability of vanadium species under water splitting conditions at high pH (refs 8, 15).

### *Ex-situ* microscopy study of photochemical corrosion

**Figure 4:**
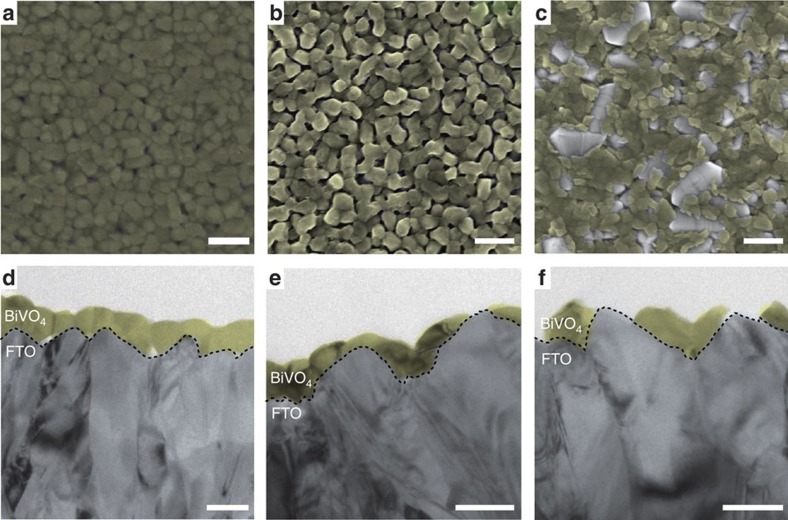
SEM and TEM characterization. SEM plan view (scale bar, 500 nm) and TEM cross-section (scale bar, 100 nm) images of pristine (**a**,**d**), degraded at pH 6.8 (**b**,**e**) and at pH 12.3 (**c**,**f**) BiVO_4_ thin films. FTO (transparent blue) and BiVO_4_ (transparent yellow) are false coloured to better highlight the difference between the two layers.

The effect of photochemical degradation on the BiVO_4_ film morphology was determined by plan view SEM and cross-sectional TEM (Fig. 4). As seen in Fig. 4a, the average grain size in as-synthesized BiVO_4_ films is ∼153±25 nm. Following stability testing at pH 6.8, the grains become rounded and voids form at grain boundaries, suggesting that corrosion initiates in intergrain regions of the polycrystalline films. At high pH, rapid photocorrosion yields large regions of exposed FTO substrate, consistent with XPS observations and the higher corrosion rate determined by ICP-MS and RBS.

TEM images of pristine BiVO_4_ show a continuous layer of ∼54±13 nm, in good agreement with the thickness determined by RBS. After stability testing at near-neutral pH for 1 h, the BiVO_4_ layer remains largely continuous but its thickness is reduced. Inspection of the cross-sectional micrographs reveals evidence of enhanced corrosion rates near the apices and reduced corrosion rates near the valleys of the film. The overall film thickness decreases to 48±13 nm. At high pH, evident signs of degradation appear after stability testing for 20 min. In apex regions of the substrate, complete loss of BiVO_4_ is observed, whereas thick islands of BiVO_4_ remain in valleys.

### *In-situ* evaluation of chemical stability

**Figure 5:**
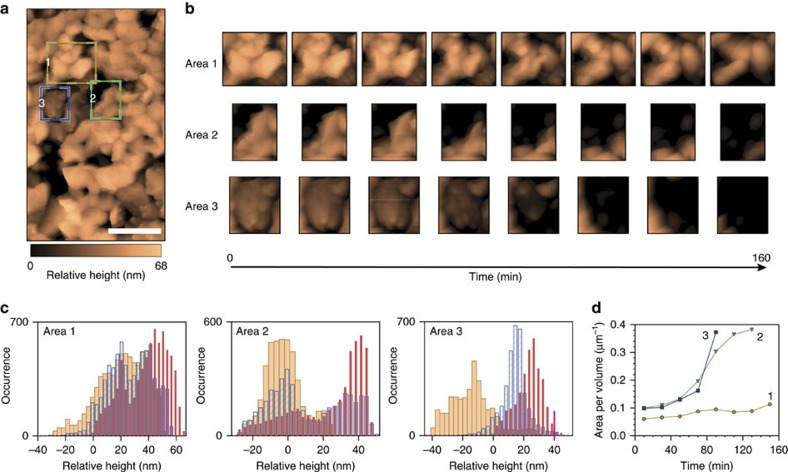
Monitoring corrosion via *in situ* EC-AFM. (**a**) EC-AFM scan (527 × 898 nm^2^; scale bar, 200 nm). The reported height is relative to a point on the underlying FTO substrate that is resolvable in all EC-AFM images. Common points have been used to align eight subsequent 1 × 1 μm^2^ scans of the same region, to correct for sample drift and rotation. Three coloured boxes indicate Regions 1 (yellow), 2 (green) and 3 (blue), whose temporal evolution were tracked in detail. (**b**) Aligned EC-AFM scans in the three regions indicated in **a** were used to monitor corrosion-induced changes to BiVO_4_ morphology at 20 min increments in 1 M KPi (pH 12.3). (**c**) Histograms showing height distributions in each of the three regions at the 10 (red)-, 70 (blue)- and 150 (orange)-min marks demonstrate that the heights of the BiVO_4_ film decreases over time. Height values <0 nm correspond to the underlying FTO substrate. (**d**) Progression of the surface area to volume ratio for region 1 (yellow), 2 (green) and 3 (blue) over the course of the 160-min test.

Although poor stability would adversely affect the performance of an operating solar fuel device, identification and enhanced understanding of the morphological, compositional and electronic factors that contribute to corrosion is required to develop strategies for improving durability and efficiency^23^,^24^,^25^. Beyond BiVO_4_, such mechanistic insights are valuable for evaluating (photo)chemical susceptibilities of other transition metal oxides and approaches to designing next-generation materials that overcome present stability limitations. Therefore, to gain more information on the corrosion mechanism, *in situ* degradation was monitored by EC-AFM. A bias of 1.23 V versus RHE was applied to the BiVO_4_ working electrode in the electrolyte solution (1 M KPi at pH 12.3) in dark, while monitoring the changes in morphology over time. A total of eight AFM topography scans were collected in the same region, to examine the degradation process over a period of 160 min (Fig. 5a,b and Supplementary Movie 1). Specifically, three different regions within the same scanned area (Fig. 5a) were selected for evaluation of variations in height and surface-to-volume ratio (Fig. 5c,d), as well as the distribution of the height profile. Close inspection of these images reveals that degradation occurs approximately uniformly at crystallographic facets exposed to the electrolyte.

Region 1 exhibits three surfaces (solid/liquid interfaces) in the upper, side and bottom part of the frame that are exposed to the electrolyte. Notably, etching progresses approximately uniformly from all three solid/liquid interfaces. In addition, the height histogram from Region 1 reveals that simultaneous etching occurs from the exposed top surface. In contrast, Region 2 has just one solid/liquid interface, which runs diagonally across the upper left portion of the region, exposed directly to electrolyte. Importantly, solid/solid boundaries that are present within this region do not appear to be specific sites for chemical attack. Analysis of the corresponding height histogram supports this interpretation, in which an approximately bimodal distribution is observed, with low and high values corresponding to the substrate and the BiVO_4_ grain, respectively. Region 3 exhibits no solid/liquid interfaces and is instead characterized by the presence of solid/solid grain boundaries, along with the exposed top surface of the central grain. Thus, this region is more protected from chemical attack and corrosion occurs almost exclusively from the top surface. As corrosion propagates from the top surface, the distribution of heights shifts monotonically towards smaller values.

Based on these *in*-*situ* EC-AFM measurements, we can conclude that corrosion of the material initiates predominantly from solid/liquid interfaces. We find no indication for selective etching of localized regions, such as at secondary phases at grain boundaries, or undercutting of the film at its interface to the FTO substrate. However, as these polycrystalline films are not completely compact, the evolution of morphology depends strongly on local geometry. Voids in intergrain regions act as sites for more rapid chemical attack, whereas adjacent grains in intimate physical contact with one another are partially protected. For those regions that are characterized by multiple solid/liquid interfaces, increases of the microscopic surface to volume ratio as etching progresses could lead to accelerated corrosion (Fig. 5d). We note that further advancement of the EC-AFM, currently underway in our laboratories, will allow for implementation of light illumination of the sample under operating conditions. However, the current time-lapse testing procedure enabled *in situ* monitoring of changes over a time scale compatible with the scan rate used for EC-AFM (Supplementary Movie 1).

### Computational evaluation of (photo)chemical corrosion

**Figure 6:**
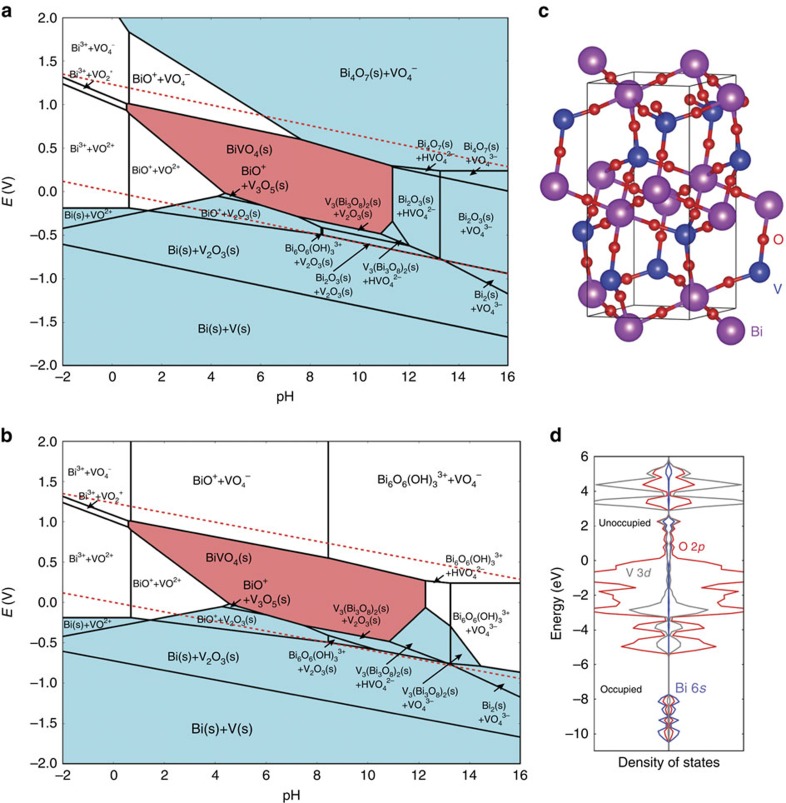
Evaluation of illuminated and dark stability by computational methods. (**a**) The Materials Project Pourbaix diagram of 50–50% Bi–V system in aqueous solution, assuming a Bi ion concentration at 10^−5^ mol kg^−1^ and a V ion concentration at 10^−5^ mol kg^−1^, and (**b**) a Pourbaix diagram with Bi_2_O_3_ and Bi_4_O_7_ phases removed. The upper red dashed line represents the potential for the oxygen evolution reaction (OER), whereas the lower red dashed line corresponds to the potential for the hydrogen evolution reaction (HER). The cyan regions denote stable solid compounds, while the pink region is BiVO_4_. In the white regions, only ions are stable in aqueous solution. (**c**) Illustration of predicted BiVO_5_ structure where blue, magenta and red spheres denote the V, Bi and O atoms, respectively. Thermodynamic evaluation of the stability of this hypothetical compound, which consists of lattice Bi^5+^ and V^5+^, is used to predict the effect of surface accumulation of photogenerated holes in BiVO_4_. (**d**) Atom projected density of states of BiVO_5_. The energy of the Fermi level is set to 0 eV.

Computational methods are employed to interpret our experimental results and they provide insight into the mechanism of corrosion under dark and illuminated conditions. We first consider the thermodynamic stability of BiVO_4_ under dark conditions at the open circuit potential, where we observe slow but progressive dissolution of films at all pH values. To this end, we have calculated Bi-V Pourbaix diagrams with ion concentrations of 10^−5^ mol kg^−1^, as implemented in the Materials Project^26^ using the hybrid calculated-experimental Pourbaix formalism approach based on the work of Persson *et al*.^27^. To the best of our knowledge, an experimental Pourbaix diagram for BiVO_4_ has not been established. However, we can gain insight into the processes responsible for photochemical and chemical corrosion of BiVO_4_ by considering equilibrium thermodynamic stability against decomposition into any combination of solid and/or aqueous species. As shown in Fig. 6a, BiVO_4_ is stable in a large region with pH values from 1 to 11 and at the electrochemical potentials within the water stability region. Unfortunately, at potentials close to the oxygen evolution potential, BiVO_4_ is no longer thermodynamically stable. Its constituent V is expected to preferentially dissolve as VO_4_^−^ ions. This is consistent with the single-element V Pourbaix diagram, where solid phases are dissolved and only VO_4_^−^ ions exist in the same region.

From a thermodynamic perspective, it is expected that the BiVO_4_ would self-passivate via formation of a chemically stable bismuth oxide at its surface. However, although our XPS experiments indicate some Bi enrichment at the surface following extended dark stability testing, we also detect the presence of Bi ions in the supporting electrolyte and conclusively observe that bulk dissolution of the BiVO_4_ occurs and self-passivation does not impede corrosion. This experimental result indicates that the thermodynamic equilibrium between the solid and liquid is not established. Indeed, a self-passivated surface of BiVO_4_ would require formation of a more stable Bi–O surface phase (for example, Bi_4_O_7_ as seen at higher potentials and pH). However, the vanadium to bismuth content in BiVO_4_ is 1:1, which means that 50% of the cations will dissolve, rendering the resulting Bi–O framework highly defective and unstable. Formation of a stable bismuth oxide or hydroxide would require significant structural rearrangement, which may be kinetically hindered under room-temperature aqueous conditions. Given these considerations, we can visualize the scenario in which structural rearrangement to form a passivating oxide on the solid surface is kinetically unfavourable by artificially removing all solid Bi-oxide phases above the OER potential (in this case, Bi_4_O_7_ and Bi_2_O_3_) from the set of phases included in the construction of the Pourbaix diagram. The result is shown in Fig. 6b, where we observe that only ions exist in the aqueous solution in the entire pH range above the OER potential. This result reveals that thermodynamic considerations cannot describe the observed dark stability of BiVO_4_ thin films. Rather, kinetic limitations associated with room-temperature structural rearrangements of corrosion products must be considered. Such a mechanism for kinetically inaccessible self-passivation is expected to be increasingly important for complex, multi-component oxides. Computational evaluation of possible kinetically hindered surface transformations may be essential for predicting the real world stabilities of novel compounds and for designing strategies for overcoming chemical instabilities.

In addition to the dark chemical instability of BiVO_4_ thin films, our experimental results indicate that dissolution of the material in water is accelerated by illumination, as well as anodic biasing to 1.23 V versus RHE. These findings suggest that accumulation of photogenerated holes at the surface of BiVO_4_ promotes photocorrosion. Furthermore, we note that previous studies have found that integration of a catalyst on the surface can suppress photocorrosion and enhance long-term stability of BiVO_4_ (refs 8, 15, 16, 19, 28, 29, 30, 31), even for the case of porous and ion-permeable catalysts, such as CoPi and FeO(OH), which do not physically isolate the semiconductor from the electrolyte environment. This suggests that efficient extraction of photogenerated holes from the near-surface region reduces chemical attack of the material.

Given these observations of photoinduced chemical instability, we consider the role of surface-accumulated holes under photoexcitation, as well as the strongly oxidative conditions present with the production of reactive oxygen species (for example, OH^·^ and O^−^)^32^,^33^, on the stability of BiVO_4_. The valence band maximum, where photogenerated holes accumulate, consists of considerable Bi 6*s* orbital character, hybridized with O 2*p* (ref. 7). In prior theoretical work, Kweon and Hwang^34^,^35^ showed that large polaron holes are the stable positive charge carriers within the monoclinic phase of this material, and that, at the surface, holes localize to BiO_6_ polyhedra, with 22% of hole charge density on Bi cations and an average of 8% of hole charge density on each of the six O anions in the polyhedron, with balance of charge located outside of the polyhedron. Therefore, we first consider the impact of the modified charge density around Bi ions due to hole localization on stability. To this end, we hypothesize the scenario that brings Bi^3+^ to Bi^5+^+2e^−^ in BiVO_4_, as Bi^5+^ is a potentially stable oxidation state for Bi, whereas Bi^4+^ is uncommon^36^,^37^. To obtain a plausible structure representing a Bi^5+^/V^5+^ compound or film, we use the Materials Project structure predictor^38^,^39^ to predict a new oxide compound containing only Bi^5+^ and V^5+^ valence states. By calculating the stability of an optimized bulk Bi^5+^ and V^5+^ oxide, we can obtain a qualitative picture of the stability of such a configuration in an oxide framework. The structure predictor algorithm searches for the most suitable structure among all known structures found in the ICSD^40^ for the target ions and valence states (in this case, Bi^5+^ and V^5+^) through a data-mining approach^38^,^39^. The resulting structure is shown in Fig. 6c. From our calculations, the projected density of states show that the Bi 6*s* orbitals are very strongly hybridized with the O 2*p* orbitals, both in the valence band and conduction band (Fig. 6d). Considering that the magnetic moment is zero in the system, it is confirmed that Bi and V in BiVO_5_ are both in 5+ oxidation states. A new phase diagram for the Bi–V–O system is generated (Supplementary Fig. 7) and it is found that BiVO_5_ is unstable by 0.215 eV per atom towards oxygen release. This large above-hull energy, defined as the energy of unstable phases, strongly indicates that the thermodynamic driving force for BiVO_5_ to decompose into BiVO_4_ and ½ O_2_ is significant. One of the reasons for the instability of BiVO_5_ is that the small ionic radius of Bi^5+^ is not compatible with the BiO_8_ structure motif in the crystal. These results suggest that any photo-induced oxidation event on Bi is likely to further destabilize the compound and increase the rate of corrosion under operating conditions, due to an effective Bi–O bond strain in the presence of Bi^5+^.

In addition to hole localization on Bi ions, we also consider the impact of oxidation of O^2−^ to O^−^ at the surface of BiVO_4_. Such a process has been implicated in the photocorroison mechanism of, for example, ZnO, where it has been proposed that degradation proceeds through hole trapping at oxygen surface sites to yield formation of O^−^ intermediate that results in irreversible loss of oxygen from the lattice as O_2_ and consequent release of Zn^2+^ ions in the solution^33^. Although the charge density of surface-accumulated holes has been predicted to be higher at Bi cations than O anions, such a mechanism is not precluded. Furthermore, the energetic position of the valence band maximum of BiVO_4_, at ∼7.27 eV below the vacuum level, renders formation of radical OH^·^ species, as found by Bard and colleagues^33^. Participation of such radicals in the oxygen loss processes described above may serve to further accelerate photocorrosion, in particular at high pH.

## Discussion

We demonstrate that polycrystalline BiVO_4_ thin films are susceptible to chemical and photochemical corrosion. Indeed, chemical attack of the semiconductor is observed under all aqueous testing conditions and is accelerated by illumination, increasing pH and applied anodic bias. Although RBS reveals near-stoichiometric composition in the bulk of as-deposited thin films, XPS indicates a non-stoichiometric composition at the surface that is affected by exposure to electrolyte. Observation of film dissolution in real time by EC-AFM indicates that chemical attack propagates approximately uniformly from exposed solid/liquid interfaces. Therefore, there is no indication of preferential chemical attack at specific regions or at crystal facets of grains, but consumption rate is dependent on local solid/liquid interface morphology. In contrast to thermodynamic predictions, self-passivation of BiVO_4_ by the formation of a chemically stable Bi oxide surface phase is not observed and bulk dissolution of films occurs on extended exposure to aqueous electrolytes. We attribute the bulk chemical instability to kinetic factors that limit room-temperature structural transformation of the V-deficient degradation product into a stable Bi oxide phase. In addition, photoexcitation results in accumulation of holes at the surface of BiVO_4_ that destabilize the lattice and increase the dissolution rate.

Mechanistic insights into the chemical and photochemical instability of BiVO_4_ can be used to guide approaches to stabilization and aid the search for active catalysts and functional interfaces that can improve durability for sustained operation. For example, efficient transfer of holes to catalyst on the surface will dramatically reduce the corrosion rate. However, unless this catalyst layer is dense and impermeable, long-term durability may be limited by the native dark chemical stability of the material and self-passivation cannot be assumed. We note that introduction of surface layers that reduce the corrosion rate and enable time for structural reorganization of the surface before dissolution might promote self-passivation and kinetically stabilize the material. Beyond BiVO_4_, the methodology presented here can be applied for rigorous investigation of the stabilities of new and emerging functional materials under operating conditions. Identification of kinetic limitations to self-passivation points to a new set of factors that should be considered in the experimental and computational search for stable, visible-light-absorbing semiconductors for photoelectrochemical energy conversion.

## Methods

### Fabrication of BiVO_4_ thin film photoelectrodes

Spin-coated bismuth vanadate thin films were prepared using a procedure adapted from the literature^6^. FTO films with nominal resistivity of ∼13 Ω/□ (Sigma Aldrich) on 10 × 10 cm^2^ glass substrates were thoroughly washed with isopropanol, detergent (Alconex) in deionized water and pure deionized water, dried with a nitrogen gun and treated for 10 min with an ozone cleaner (Jelight Model 42) before deposition of BiVO_4_ by spin coating. In a typical deposition, 15 ml of a 0.2 M solution of bismuth (III) nitrate pentahydrate (Sigma Aldrich, ≥98%) in acetylacetone (Sigma Aldrich, ≥99%) and 100 ml of a 0.03-M solution of vanadium(IV)-oxy acetylacetonate in acetylacetone were prepared separately and sonicated for 10 min. Then, the two solutions were mixed together and sonicated for an additional 5 min. Approximately 1–1.2 millilitres of the resulting solution millilitres (enough solution to homogeneously cover the whole surface) was filtered with 0.45 μm nylon filters (Thermo Fisher Scientific) and dispensed onto the 10 × 10 cm^2^ FTO/glass slide. The substrate was then spun twice in a row at 1,000 r.p.m. for 6 s on a spin coater (Laurell Technologies) with an acceleration rate of 150 r.p.m.  s^−1^. After this spin-coating cycle, the substrate was annealed for 10 min in air at 500 °C in a muffle furnace (Cole-Parmer). This procedure (that is, spin coating followed by short annealing) was repeated nine times. After the last spin-coating cycle, the substrate was annealed for 2 h at 500 °C to achieve a final thickness of ∼50 nm.

BiVO_4_ thin film photoelectrodes were prepared by dicing the BiVO_4_-coated FTO/glass into ∼1 cm × 1.3 cm pieces. To expose FTO to provide electrical connection to external leads, an ∼0.3-cm-wide strip of the BiVO_4_ thin film along the long side of the electrode was etched away using 0.1 M HCl (Sigma Aldrich, ACS reagent, 37%). A Cu wire was connected to the exposed FTO using silver conductive epoxy (Circuit Works, CW2400) and the electrode with the wire was dried for 20 min at 60 °C. Then, the wire was isolated with a glass tube and the Cu wire, silver epoxy and exposed FTO were all sealed together with the glass tube using a non-conductive aqueous resistant epoxy (Loctite 615 Hysol). Exposed surface areas of the assembled photoelectrodes were determined by optical scanning and digital image analysis with Image J. Typical areas were in the range of 0.5–1.0 cm^2^.

### Photoelectrochemistry and stability measurements

All photoelectrochemical measurements were performed in a three-electrode photoelectrochemical cell with a planar quartz window (5 cm^2^) using a BioLogic SP200 potentiostat. Bismuth vanadate photoanodes were configured as the working electrodes, a coiled Pt wire was used as counter electrode and a Ag/AgCl (3 M NaCl, BASI) served as reference electrode. Measurements were performed in electrolyte consisting of 1 M KPi buffer solutions at pH 6.8 or 12.3 with or without 0.1 M sodium sulfite as sacrificial reagent, depending on the particular experiment. In detail, 1 M KPi at pH 6.8 was prepared from KPi monobasic (Sigma Aldrich, ≥99%, 34.0 g, 0.25 mol) and KPi dibasic (Sigma Aldrich, ≥98%, 43.5 g, 0.25 mol) in 500 ml of water. Likewise, 1 M KPi at pH 12.3 was prepared using KPi dibasic (43.5 g, 0.25 mol) and KPi tribasic (Sigma Aldrich, ≥98%, 53.1 g, 0.25 mol) in 500 ml of water. The diffusion constants for HPO_4_^2−^ and PO_4_^3−^ are reported in Supplementary Note 1 (ref. 41). To evaluate the performance of photoanodes as semiconductor light absorbers, sodium sulfite (Sigma Aldrich, ≥98%, 6.3 g, 0.05 mol) was added to buffer solutions as a sacrificial hole acceptor. For material degradation studies, measurements were performed in the absence of sodium sulfite. Unless otherwise noted, all measurements under illumination were performed using simulated AM 1.5 light (Solar Light) adjusted to 100 mW cm^−2^ using a calibrated Si photovoltaic cell (SolarSim calibration, Newport).

Systematic evaluations of performance and stability at pH 6.8 and 12.3 were carried out using the following procedure. First, baseline photoelectrochemical performance was established by running CVs from the open circuit potential (*E*_oc_) to 1.8 V versus RHE at a scan rate of 100 mV s^−1^ in the presence of 0.1 M sodium sulfite as sacrificial reagent. Both dark and illuminated CVs were collected. It is noteworthy that for the sake of clarity, only anodic sweeps obtained from these CVs are shown in Fig. 1. Complete CVs are shown in Supplementary Fig. 4. Second, stability of the same electrodes was tested by performing chronoamperometry in pure 1 M KPi buffer solutions (pH 6.8 or 12.3) in the dark or under illumination at *E*_oc_ or at 1.23 V versus RHE. Fresh electrodes were used for each of the illumination and pH conditions. Stability tests at pH 6.8 were run for 60 min, whereas those at pH 12.3 were run for 20 min due to the more rapid degradation. Finally, the post-degradation performance was evaluated by collecting CVs using conditions identical to those used for baseline performance evaluations.

### Material characterization

Baseline X-ray diffraction characterization of FTO/glass substrate coated with a spin-cast BiVO_4_ thin film was obtained with a Rigaku SmartLab X-ray diffractometer. X-ray diffraction patterns were collected with a Cu source at 0.5° with respect to the sample and parallel beam optics. To provide insights into the mechanism and consequences of BiVO_4_ degradation under photoelectrochemical conditions, comparative measurements before and after stability testing were performed using a variety of complementary spectroscopic and microscopic methods. To evaluate morphological changes, SEM was performed on a Zeiss Gemini Ultra-55 analytical field emission SEM and on an FEI Quanta 250 FEG. In addition, cross-sectional TEM images were obtained on an FEI Tecnai F30, with a 300-kV acceleration voltage.

The near-surface chemical composition and valence band structure were determined by XPS using a monochromatized Al Kα source (hν=1486.6 eV), operated at 225 W, on a Kratos Axis Ultra DLD system at a takeoff angle of 0° relative to the surface normal and a pass energy for the narrow scan core level and valence band spectra of 20 eV. Spectral fitting was conducted using Casa XPS analysis software. Spectral positions were corrected using adventitious carbon by shifting the C 1*s* core level position to 284.8 eV and curves were fit with quasi-Voigt lines following Shirley background subtraction.

Complimentary bulk composition measurements were obtained by RBS, which also allowed for determination of nominal film thickness. He^+^ ions were accelerated to 3,040 keV on a 5SDH pelletron tandem accelerator manufactured by National Electrostatics Corporation with a sample tilt of 30–60° and backscattered ions were detected at an angle of 165° using a Si surface barrier detector. Data fitting was performed with SIMNRA software. RBS analysis may allow for calculation of an effective film thickness, assuming a planar and homogeneous BiVO_4_ overlayer deposited on a flat surface. Although our films exhibit good coverage with a reduced roughness on the surface, the underlying FTO has a similar roughness and we have to account that the model is affected by these parameters.

Analysis of corrosion products in electrolyte solution was conducted by ICP-MS with an Agilent 7,900 using a standard configuration consisting of Micromist nebulizer and quartz sample introduction system of Scott-type with spray chamber and one piece torch with a 2.5-mm inner diameter injector; Ni cones were used throughout. Internal standards were added on-line via the sample delivery peristaltic pump. The instrument was optimized using default autotune conditions directly from the MassHunter Workstation for U-HMI. Samples were measured in helium collision mode for all analytes.

EC-AFM was performed on a Bruker Dimension Icon AFM system, using an *in situ* electrochemical cell as sample holder, equipped with a BiVO_4_ working electrode, a coiled Pt wire as counter electrode and a Ag wire as pseudo-reference. All the measurements were performed in pH 12.3 and a BioLogic SP200 potentiostat was used to apply a 1.23 V versus RHE bias. Topography AFM images were acquired in Scanasyst mode with Si tips (Bruker Scanasyst—Air). It is worth noting that no degradation of the Si tip was observed over the measurement period.

### Evaluation of illuminated and dark stability by theoretical methods

The first-principles calculations are based on density functional theory using the Vienna software package (VASP)^42^. We choose the PAW pseudopotentials^43^ with the generalized gradient approximation as implemented by Perdew, Burke and Ernzerhoff (PBE^44^). The PBE+U method is used to address the on-site Coulomb interactions in the localized *d* orbitals by adding an additional Hubbard-type U-term, with the value of 3.25 eV for V as reported for binary metal oxides^44^. A 6 × 6 × 6 Monkhorst–Pack *k*-point mesh for the integrations over the Brillouin zone is used. The energy cutoff is 450 eV and the spin polarization is included in all calculations.

The analysis of the Bi-V ternary Pourbaix diagram is based on the work of Persson *et al*.^46^, where solid and dissolved species are combined in a single-phase diagram to determine the stable species (solids and/or aqueous ions) as a function of pH and potential^36^. Following ref. 46, the theoretical data obtained with the PBE+U method are used for the total energies of solid phases and the experimental data are used for the dissolved ions.

To construct the phase diagram, we use the phase diagrams app^47^ from the Materials Project database. By calculating the energies of all known compounds in a given chemical system (for example, Bi–V–O), we can determine the phase diagram for that system at 0 K and 0 atm.

### Data availability

The source data that support the findings of this study are included in the Article, Supplementary Information and Supplementary Data 1.

## Additional information

**How to cite this article:** Toma, F. M. *et al*. Mechanistic insights into chemical and photochemical transformations of bismuth vanadate photoanodes. *Nat. Commun.* 7:12012 doi: 10.1038/ncomms12012 (2016).

## Supplementary Material

Supplementary Information Supplementary Figures 1-7, Supplementary Tables 1-2, Supplementary Note 1 and Supplementary Reference

Supplementary Movie 1 Direct observation of bismuth vanadate corrosion by *in situ* EC-AFM. This movie shows time-lapse degradation of a bismuth vanadate (BiVO_4_) thin film deposited on fluorine-doped tin oxide (FTO), as reported in Figure 5. The measurements were performed in 1 M KPi with an applied bias of 1.23 V vs RHE under dark conditions. Aligned EC-AFM scans were used to monitor BiVO_4_ corrosion over a total time frame of 160 minutes.

Supplementary Data 1 Source data used to generate figures.

## Figures and Tables

**Table 1:** Bulk and surface composition analysis.

	Testing condition	Bi (at.%)	V (at.%)	Thickness* (nm)
Ideal†		16.7 (1)	16.7 (1)	
RBS	Pristine	14.3±0.5	14.5±1.3	51.6±3.2
	pH 6.8	14.5±0.7	14.1±0.7	44.1±3.7
	pH 12.3	11.1±1.5	11.1±1.5	37.4±1.8
XPS	Pristine	23.5±1.5	16.3±0.7	
	pH 6.8	25.3±0.7	15.4±0.4	
	pH 12.3	26.7±1.6	14.0±0.5	

RBS, rutherford backscattering spectrometry; XPS, X-ray photoelectron spectroscopy.

^*^Thickness calculated from the number of atoms detected by RBS, where bulk density of BiVO_4_ is assumed^48^. Tests were performed for 60 min at pH 6.8 and 20 min at pH 12.3.

^†^Ideal atomic ratio and ideal stoichiometric ratio in parenthesis. Composition analysis including O at.% is reported in Supplementary Table 1.

**Table 2:** ICP-MS analysis of electrolyte following stability testing.

pH	Light condition	Bias* (V versus RHE)	Time (min)	Degradation rate on V basis (nm min^−1^)	Degradation rate on Bi basis (nm min^−1^)
6.8	Light	1.23	60	0.125	0.122
	Dark	1.23	60	0.03	0.04
	Light	*E*_oc_	60	0.06	0.05
	Dark	*E*_oc_	60	0.1	Not detectable
12.3	Light	1.23	20	1.24	1.32
	Dark	1.23	20	0.17	0.14
	Light	*E*_oc_	20	0.69	0.71
	Dark	*E*_oc_	20	0.26	0.21

ICP-MS, inductively coupled plasma mass spectrometry, *E*_oc_, open circuit voltage; RHE, reversible hydrogen electrode.
