# Supplementary material for: Mechanistic insights into chemical and photochemical transformations of bismuth vanadate photoanodes
Source: Nat Commun. 2016 Jul 5;7:12012. doi: 10.1038/ncomms12012 (PMC4935965; doi:10.1038/ncomms12012)
Supplement: Supplementary Information — Supplementary Figures 1-7, Supplementary Tables 1-2, Supplementary Note 1 and Supplementary Reference [file ncomms12012-s1.pdf]

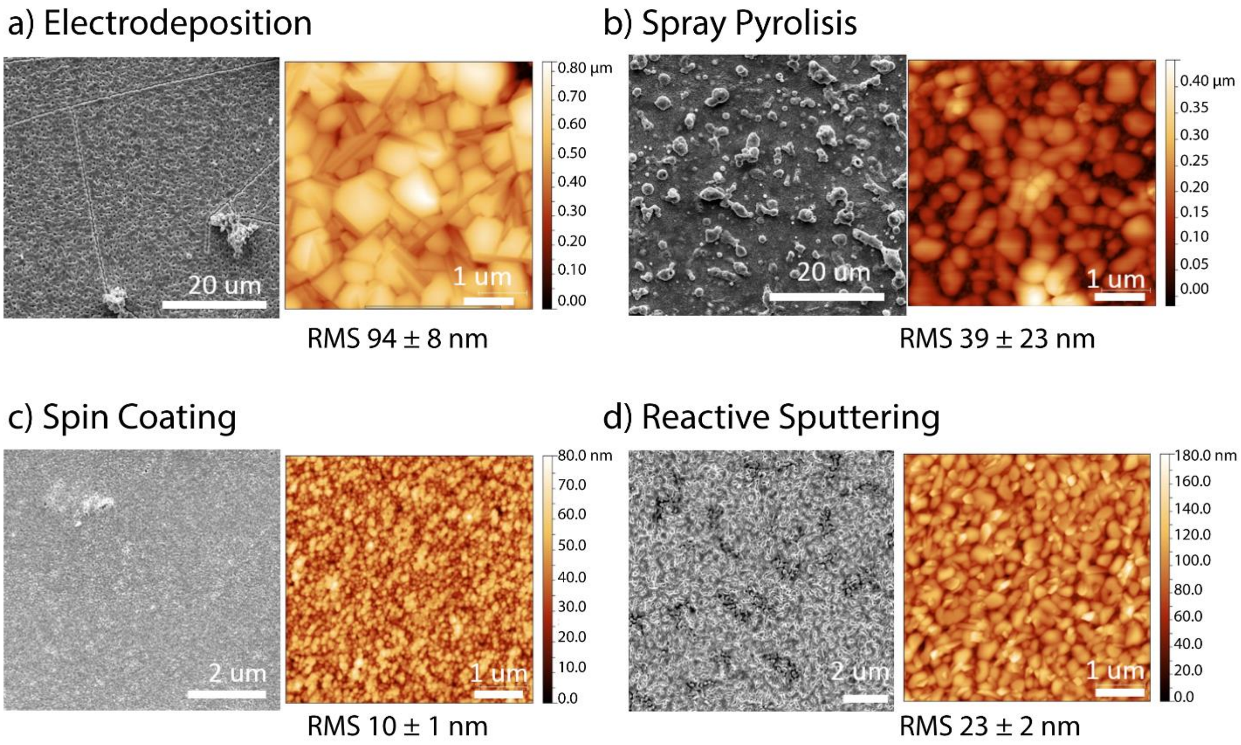

Supplementary Figure 1: SEM (left) and AFM (right) images of different $BiVO_4$ samples deposited by a) electrodeposition (Mo:$BiVO_4$), b) spray pyrolysis (W:$BiVO_4$), c) spin coating ($BiVO_4$), d) reactive sputtering ($BiVO_4$).

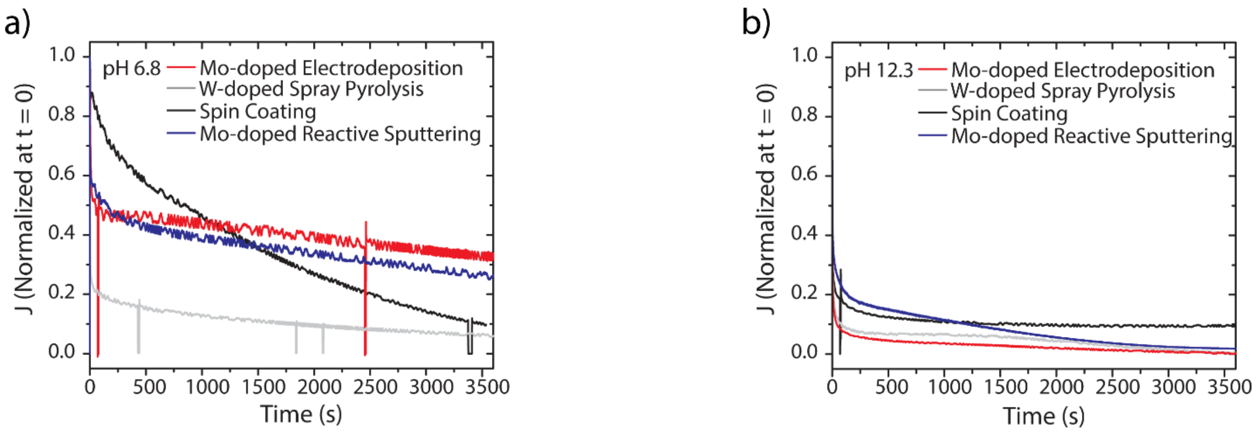

Supplementary Figure 2: Chronoamperometry curves of $BiVO_4$ thin films at a) pH 6.8 and at b) pH 12.3. Photocurrent is normalized at t = 0 s and all the traces show photocurrent decline over time.

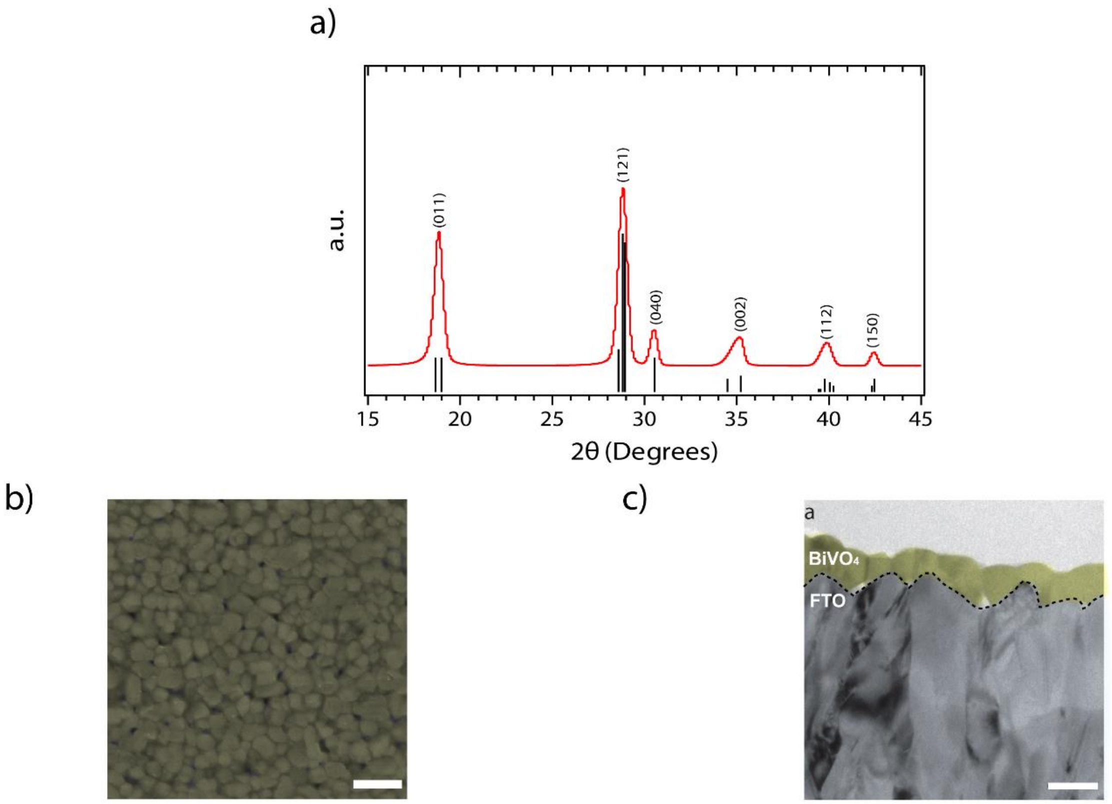

Supplementary Figure 3: a) XRD pattern, b) SEM plan view (scale bar 500 nm), and c) TEM cross section (scale bar 100 nm) images of pristine $BiVO_4$. The XRD pattern matches with the desired monoclinic $BiVO_4$ phase (black lines, space group I2/b, JPCDS no. 04-010-5711).

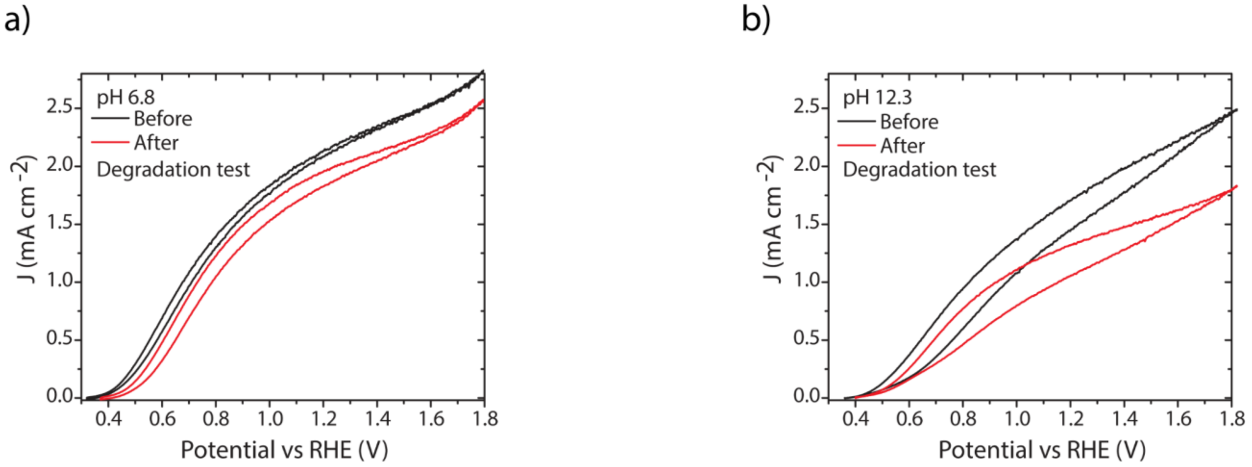

Supplementary Figure 4: Typical cyclic voltammogramms of $BiVO_4$ thin films in the presence of Na_2SO_3 sacrificial reagent, at a) pH 6.8 and at b) pH 12.3 before (black) and after (red) degradation in the corresponding phosphate buffer.

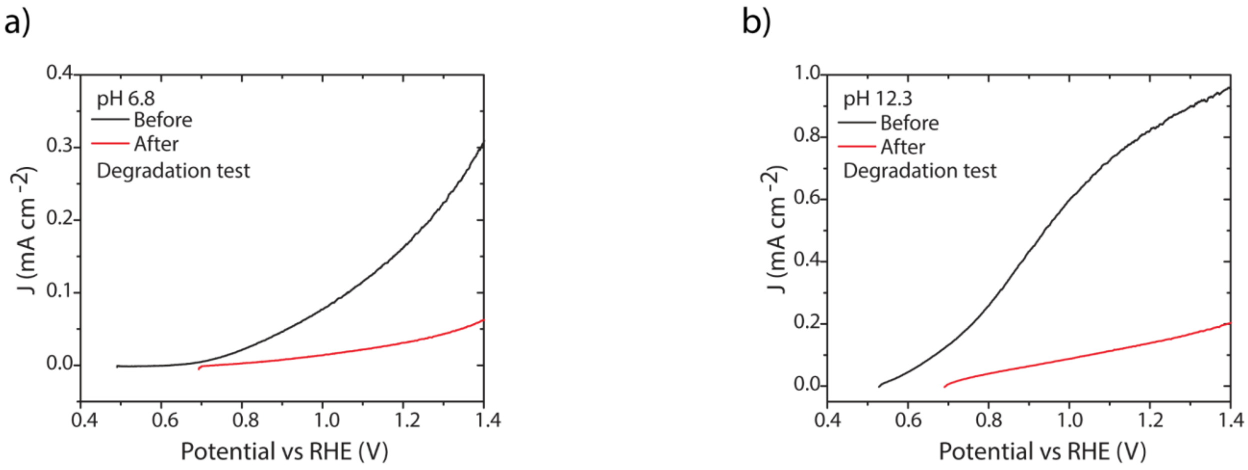

Supplementary Figure 5: Typical cyclic voltammogramms of $BiVO_4$ thin films, at a) pH 6.8 and at b) pH 12.3 before (black) and after (red) degradation in the corresponding phosphate buffer.

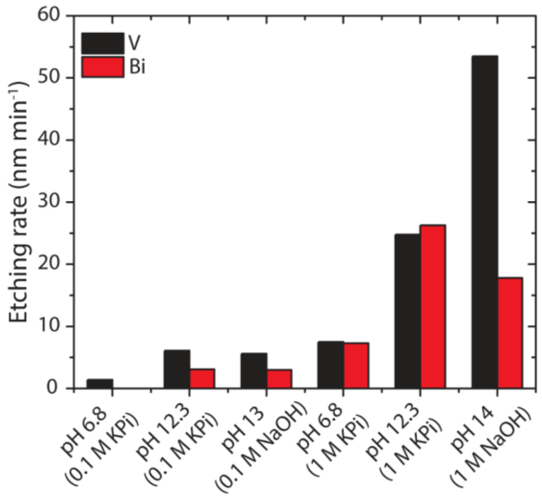

Supplementary Figure 6. ICP-MS data column plot of etch rate determined on the basis of V (black) and bismuth (red) loss to solution at 1.23 V vs. RHE under illumination in different electrolytes.

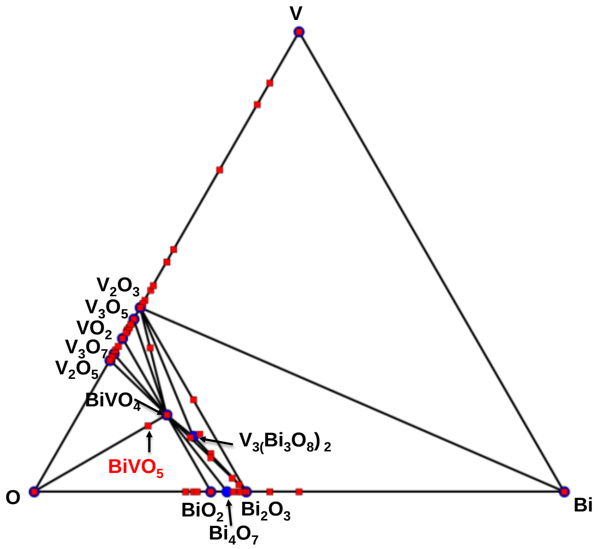

Supplementary Figure 7: Phase diagram of $BiVO_5$. The calculated phase diagram of Bi-V-O system. Blue circles indicate the stable compounds while red squares indicate the unstable compounds. All stable compounds are labeled in black while only one unstable compound ($BiVO_5$) is marked in red in the phase diagram.

Supplementary Table 1: Bulk (RBS) and surface (XPS) composition analysis at 1.23 V vs. RHE					
	**Testing Condition**	**Bi (at. %)**	**V (at. %)**	**O (at. %)†**	**Thickness* (nm)**
**Ideal****		16.7 (1)	16.7 (1)	66.6 (4)	
**RBS**	Pristine	14.3 ± 0.5	14.5 ± 1.3	71.1 ± 1.5	51.6 ± 3.2
	pH 6.8 (Light)	14.5 ± 0.7	14.1 ± 0.7	71.4 ± 1.2	44.1 ± 3.7
	pH 6.8 (Dark)	14.1 ± 0.2	13.6 ± 0.3	72.3 ± 0.1	51.8 ± 1.9
	pH 12.3 (Light)	11.1 ± 1.5	11.1 ± 1.5	77.8 ± 3.0	37.4 ± 1.8
	pH 12.3 (Dark)	14.0 ± 1.4	13.5 ± 2.1	72.5 ± 35	48.8 ± 1.7
**XPS**	Pristine	23.5 ± 1.5	16.3 ± 0.7		
	pH 6.8 (Light)	25.3 ± 0.7	15.4 ± 0.4		
	pH 6.8 (Dark)	25.6 ± 0.8	15.6 ± 0.1	58.7 ± 1.0	
	pH 12.3 (Light)	11.1 ± 1.5	11.1 ± 1.5		
	pH 12.3 (Dark)	25.3 ± 0.5	11.5 ± 0.9	63.2 ± 1.4	

† The RBS-determined compositions include values for oxygen content. Notably, these values are determined assuming a closed and homogeneous two layer model with roughness. Significant error may be present in the oxygen composition values from RBS due to film non-idealities and the presence of oxygen in both substrate and film. Values are given here for completeness, and only Bi/V ratios should be considered as quantitative for this case.

Supplementary Table 2: Summary of ICP-MS data

Conditions	Area (cm^2)	Soaking Time	Volume of solution (mL)	Concentration of V [ppb]	Degradation rate on V basis (nm min^{-1})	Concentration of Bi [ppb]	Degradation rate on Bi basis (nm min^{-1})
0.1 M KPi pH 6.8, dark	1.084	72 h	40	15.4860	0.00121	11.7251	0.0002
0.1 M KPi pH 6.8, 1.23 V vs RHE, light	0.744	60 min	26	4.3462	0.0231	<0.000	0
0.1 M KPi pH 12.3, dark	1.136	72 h	40	14.1717	0.0011	30.2510	0.0005
0.1 M KPi pH 12.3, 1.23 V vs. RHE, light	0.725	20 min	33	14.7263	0.3059	30.7052	0.1559
1 M KPi pH 6.8, dark	0.922	72 h	40	79.4086	0.0073	226.9524	0.0051
1 M KPi pH 6.8, 1.23 V vs. RHE, light	0.7	60 min	20	28.8774	0.1255	114.8249	0.1219
1 M KPi pH 6.8, 1.23 V vs. RHE, dark	0.621	60 min	25	8.0539	0.0493	28.3183	0.0424
1 M KPi pH 6.8, Eoc, dark	0.849	60 min	34	0.2665	0.0016	<0.000	0

1 M KPi pH 12.3, dark	0.963	72 h	40	131.3746	0.0115	439.4744	0.0094
1 M KPi pH 12.3, 1.23 V vs. RHE, light	0.693	20 min	25	75.4633	1.2424	327.4593	1.3174
1 M KPi pH 12.3, 1.23 V vs. RHE, dark	0.963	20 min	25	13.8179	0.1637	49.0137	0.1419
1 M KPi pH 12.3, Eoc, dark	0.76	20 min	32	13.5591	0.2605	44.7159	0.2099
0.1 M NaOH pH 13, dark	1.16	72 h	40	79.4982	0.00579	342.3710	0.0061
0.1 M NaOH pH 13, 1.23 V vs. RHE, light	0.731	20 min	27.5	16.4018	0.2816	35.3667	0.1484
1 M NaOH pH 14, dark	1.19	72 h	40	220.2265	0.0156	960.5204	0.01667
1 M NaOH pH 14, 1.23 V vs RHE, light	0.747	20 min	40	109.5540	2.6771	149.3423	0.8918

Supplementary Note 1:

To test the effect of buffer capacity, our measurement sequence, in the absence of sacrificial hole acceptor, was as follows: (1) measure dark CV, (2) measure illuminated CV, (3) perform chronoamperometric stability test, (4) measure dark CV after settling for 1-2 min, (5) measure illuminated CV. Therefore, we can compare the difference between initial and final CV scans with the photocurrent decline during the chronoamperometric tests. We show the anodic sweep obtained before and after degradation at pH 6.8 and pH 12.3 in phosphate buffer (also shown in Supplementary Figure 5), in which the decrease of the photocurrent at 1.23 V vs. RHE is also apparent.

The diffusion constants for HPO_4^{2-} and PO_4^{3-} are 7.59×10^{-10} m^2 s^{-1} and 6.1×10^{-10} m^2 s^{-1}, respectively, and are very similar and fast.[1] Therefore, the few minutes of settling time between the chronoamperometry (stability evaluation) and the cyclic voltammetry afterwards are sufficient to level any concentration gradients and fully replenish the diffusion layer that could potentially be depleted of OH^-.

In addition, we consider the buffer capacity of the bulk electrolyte used for the measurements. Given the 1 M buffer concentration (0.5 M $H_2PO_4^-$ and 0.5 M HPO_4^{2-} at pH 6.8 and 0.5 M HPO_4^{2-} and 0.5 M PO_4^{3-} at pH 12.3) and the current densities at which we operate, the maximum change of pH would be 0.001 and 0.0003 at the conclusion of the experiments at pH 6.8 and 12.3, respectively. This change is negligible and cannot account for the reduced photocurrent density.

For these reasons, neither local nor bulk pH changes can account for the observed behavior. Therefore, we can conclude that the changes of the native catalytic activity of the surface are responsible for much of the photocurrent decline in the chronoamperometric test, as well as the

smaller reduction of the photocurrents measured by comparing CVs in the presence of sulfite before and after the test.

SUPPLEMENTARY REFERENCES:

1 Buffle, J., Zhang, Z., Starchev, K., Metal flux and dynamic speciation at (bio)interfaces. Part I: critical evaluation and compilation of physicochemical parameters for complexes with simple ligands and fulvic/humic substances. *Environ. Sci. Technol.* **41**, 7609-7620 (2007).
